# Population pharmacokinetics and dosing optimization of imipenem in Chinese elderly patients

**DOI:** 10.3389/fphar.2024.1524272

**Published:** 2025-01-09

**Authors:** Jing Wang, Qiu Fang, Xuemei Luo, Lu Jin, Huaijun Zhu

**Affiliations:** ^1^ Department of Pharmacy, Nanjing Drum Tower Hospital, Nanjing, Jiangsu, China; ^2^ Department of Pharmacy, Nanjing Drum Tower Hospital Clinical College of Nanjing University of Chinese Medicine, Nanjing, Jiangsu, China

**Keywords:** imipenem, population pharmacokinetics, dosing optimization, elderly patients, Monte Carlo

## Abstract

**Objectives:**

To assess the pharmacokinetics and pharmacodynamics of imipenem in a retrospective cohort of hospitalized Chinese older patients.

**Methods:**

A population pharmacokinetic (PPK) model was constructed utilizing a nonlinear mixed-effects modeling approach. The final model underwent evaluation through bootstrap resampling and visual predictive checks. Additionally, a population pharmacokinetic and pharmacodynamic analysis was conducted employing Monte Carlo simulations to investigate the impact of commonly used dosing regimens (0.25 g every 6 h, 0.5 g every 6 h, 0.5 g every 8 h, 1 g every 6 h, 1 g every 8 h, and 1 g every 12 h) on the likelihood of achieving the target therapeutic outcomes.

**Results:**

A total of 370 observations available from 142 patients were incorporated in the PPK model. A two-compartment PPK model with linear elimination best predicted the imipenem plasma concentrations, with the creatinine clearance as a significant covariate of clearance. Typical estimates for clearance, inter-compartmental clearance, central and peripheral volume were 13.1 L·h^−1^, 11.9 L·h^−1^, 11.7 L, 29.3 L, respectively.

**Conclusion:**

The pharmacokinetics of imipenem in elderly patients were effectively characterized by the established PPK model, which includes creatinine clearance as a key covariate. This research will enhance our understanding of imipenem elimination and support precision dosing in this patient demographic.

## Introduction

Imipenem is a prominent member of the carbapenem class of antibiotics, recognized for its extensive antibacterial coverage against Gram-positive, Gram-negative, and anaerobic bacteria, thus widely used in the treatment of lower respiratory tract infections, abdominal infections, urinary tract infections, septicemia, infective endocarditis, reproductive system infections, bone and joint infections, as well as mixed infections and severe infections caused by unidentified pathogens (Zhanel et al., 2007; Papp-Wallace et al., 2011). Normally, it is quickly metabolized into an inactive compound by the enzyme dehydropeptidase (DHP-1) in the kidney’s brush-border, and therefore must be co-formulated with cilastatin, a DHP-1 inhibitor, which could prevent the occurrence of renal tubular necrosis and prolongs the therapeutic effect of imipenem (Koucheki et al., 2021).

Imipenem is a hydrophilic molecule with a short plasma half-life of about 1 hour and exhibits low plasma protein binding (approximately 20%). As a time-dependent antibiotic, its bactericidal activity is best measured by the pharmacokinetic/pharmacodynamic (*p*K/PD) index of maintaining free plasma concentration above the minimum inhibitory concentration of the pathogens (*f*T > MIC), aiming for at least 40% of the dosing interval (Gomez et al., 2015; Dinh et al., 2022). As reported by multiple clinical researches, it requires higher pharmacodynamic index of 100% *f*T > MIC for critically ill patients (Bai et al., 2024). However, the PK profiles of carbapenems were notably altered in elderly patients due to their altered physiological and pathological change and more frequent use of concomitant medications.

Elderly individuals are more prone to infections due to various factors such as underlying diseases (e.g., cardiovascular diseases, diabetes), polypharmacy, decreased physical function, and compromised immune system (Mangoni and Jackson, 2004; Medellín-Garibay et al., 2022). Several literature reviews have extensively documented the pharmacokinetic changes in elderly patients (Klotz, 2009; Benson, 2017). Meanwhile, there exists considerable interpatient variability in the influence of age on pharmacokinetics, leading to increased variability in these parameters compared to younger patient cohorts. As reported by Abdulla (Abdulla et al., 2021), age was found to be significantly correlated with TDM target attainment of β-lactam antibiotic therapy. This association may be related to the decline of renal function in elderly patients. However, there is limited pharmacokinetic research on the use of imipenem in elderly patients at present. Only one preliminary study (Pietroski et al., 1991) is accessible, which comprises a limited cohort of merely thirteen patients.

The main goal of this research was to evaluate the pharmacokinetic characteristics of imipenem in elderly patients through nonlinear mixed effects modeling (NONMEM). Important factors influencing changes in imipenem exposure were discovered, and suitable dosage suggestions were put forward based on the final model which might promote the judicious use of imipenem in geriatric patients.

## Materials and methods

### Study design and ethics

This research obtained authorization from the Scientific and Research Ethics Committee of Nanjing Drum Tower Hospital, the affiliated institution (No. 2023-380-02), and all methodologies were executed in compliance with ethical guidelines. We performed a retrospective observational study spanning from October 2021 to April 2024. The participants who fulfilled the inclusion criteria were as follows:1) patients aged 60 years or above, 2) patients who received imipenem and cilastatin sodium for injection and 3) plasma concentrations were monitored. The exclusion criteria were as follows: 1) incomplete clinical evaluation (unavailable data on renal function, biochemical indicators and other information; 2) patients undergoing extracorporeal membrane oxygenation (ECMO); 3) other factors deemed unsuitable for this study by the researchers.

### Data collection and sampling schedule

Demographic factors, including sex, age, and body weight, along with medication details such as dosage, dosing frequency, and administration timing, as well as laboratory results including white blood cell count (WBC), hemoglobin (HGB), platelet count (PLT), globulin (GLO), apolipoprotein A (APOA), apolipoprotein B (APOB), estimated glomerular filtration rate (eGFR), alanine aminotransferase (ALT), albumin (ALB), gamma-glutamyl transferase (GGT), aspartate aminotransferase (AST), total protein (TP), alkaline phosphatase (AKP), total bilirubin (TBIL), direct bilirubin (DBIL), and creatinine (CR) were gathered using a standardized format through the hospital’s electronic medical record system. The creatinine clearance rate (CLCR) was calculated by Cockcroft-Gault Equation.

### Quantification of imipenem concentrations

The patients were administered imipenem empirically, receiving an infusion dose ranging from 250 mg to 1000 mg with the administration intervals varied from every 6 h to every 12 h. The infusion rate was established based on the actual infusion duration documented in the Electronic Health Record. In this study, the concentrations of imipenem present in plasma were analyzed using HPLC. Sample plasma of 300 μL was mixed with 20 μL of 5-hydroxyindole-3-acetic acid (1 mg ·mL^−1^) and 100 μL of 3-morpholine propyl sulfonic acid solution (0.5 mol ·L^−1^), which was used as stabilizer. The solution was subjected to vortexing for 30 s and subsequently transferred to an ultrafiltration centrifuge tube, where it was centrifuged at 4°C for 10 min at 12,000 rpm. After centrifugation, a 30 μL aliquot of the ultrafiltrate was injected into the analytical system. The mobile phase utilized for chromatographic separation comprised methanol and 10 mmol·L^−1^ potassium dihydrogen phosphate in a ratio of 6:94 (v/v, pH 6.8), which was filtered through a 0.45 μm hydrophilic polypropylene filter. The separation was performed on a TSK gel ODS-100 V column (250 × 4.6 mm, 5 μm) maintained at a constant temperature of 30°C. The UV detector was set at 300 nm and the overall detection time was 12 min. The calibration curves demonstrated acceptable linearity over 0.5–50 μg⋅mL^−1^, with a limit of quantitation (LOD) of 0.5 μg⋅mL^−1^ for imipenem.

### Population pharmacokinetic modeling

#### Base model

The pharmacokinetic model was developed using NONMEM 7.3.0 (ICON Development Solutions, Ellicott City, MD, United States), a nonlinear mixed-effects software base model. The foundational structure of the model was explored through both one- and two-compartment models, which were assessed for their suitability based on first-order elimination kinetics following intravenous drug administration. This step was crucial for determining the most appropriate structural model for the pharmacokinetic analysis. Exponential error models were employed to define inter-individual variability (IIV, η) in pharmacokinetic parameters, which exhibited a symmetric distribution with a mean of zero and a variance of ω^2^. Furthermore, to evaluate residual variability (RSV) in the observed drug concentrations, a comprehensive analysis involving various error models was conducted. These models included exponential, additive, proportional, and combined (additive plus proportional) error models, each assuming a symmetric distribution around a mean of zero with a variance represented by σ^2^. The choice of error model plays a pivotal role in accurately describing the discrepancies between observed and model-predicted concentrations, thereby refining the pharmacokinetic model’s precision and reliability.

The formulations for these error models are as follows:

$$\text{Exponential error model}:C_{\text{obs}}=C_{\text{pred}}\times \exp^{\varepsilon }$$

$$\text{Additive error model}:C_{\text{obs}}=C_{\text{pred}}+\varepsilon$$

$$\text{Proportional error model}:C_{\text{obs}}=C_{\text{pred}}\times \left(1+\varepsilon \right)$$

$$\text{Combined error model}:C_{\text{obs}}=C_{\text{pred}}\times \left(1+\varepsilon \right)+\varepsilon^{\prime }$$

in these equations, C_obs_ represents the observed drug concentration, C_pred_ denotes the concentration predicted by the model, and ε, ε′ signify the components of residual variation.

#### Covariate screening

Prior to covariate screening, an exploratory analysis was undertaken to analyze the associations between covariate-parameter pairs. To prevent collinearity, which may destabilize the stability of the model and hinder the clarity of parameter estimate interpretations, variables exhibiting high correlation coefficients (greater than 0.5) were excluded from simultaneous inclusion in the final model (Byon et al., 2013). The stepwise method, integrating both forward inclusion and backward elimination steps, was employed to perform significant covariate screening. In the forward selection, the objective function value (OFV) had to decrease by at least 3.84 (corresponding to *p* < 0.05, chi-squared distribution with one degree of freedom) from the previous model. This threshold was chosen based on standard statistical criteria for a significant improvement in model fit (Ahamadi et al., 2019). Similarly, for backward elimination, an increase of at least 6.63 in the OFV (corresponding to *p* < 0.01, chi-squared distribution with one degree of freedom) from the previous model was required, ensuring that only covariates contributing substantially to the model were retained.

#### Model evaluation

The final model underwent assessment through a Goodness-of-Fit (GOF) analysis, which entailed graphing observed concentrations (DV) in relation to both population-predicted concentrations (PRED) and individual-predicted concentrations (IPRED) to determine the model’s predictive validity. Additionally, conditional weighted residuals (CWRES) were plotted against PRED and time from last dose to assess the residual variability and temporal patterns in model predictions, ensuring a consistent performance across the range of predicted values. Besides, a non-parametric bootstrap analysis and prediction-corrected Visual Predictive Check (pcVPC) were conducted to evaluate the stability and reliability of the final model. One thousand datasets were generated through random sampling from the original dataset, facilitating the estimation of model parameters for each. These parameters were then used to calculate median values and 95% confidence intervals (CIs), defined as the range from the 2.5th to the 97.5th percentiles. The outcomes of the bootstrap analysis were subsequently juxtaposed with the parameters from the final model to ensure consistency and reliability (Shen and Machado, 2017). A pcVPC was conducted to evaluate the model by comparing the observed concentrations with the model predictions (Bergstrand et al., 2011). Leveraging the population pharmacokinetic (PK) parameter estimates derived from the final population PK model, concentration-time profiles within the dataset were simulated across 1,000 iterations. For each time point, the 90% prediction interval of the simulated concentrations was computed and compared with the observed concentrations. Both the observed data and model predictions were normalized to the median population prediction for each bin to account for multiple covariates. The model’s performance was considered satisfactory if the observed data were sufficiently represented by the 5th, 50th, and 95th percentiles of the simulated data.

An additional cohort of 22 patients who met the inclusion criteria was retrospectively gathered and assessed to form an external validation group. The model’s predictive precision and accuracy were analyzed using a range of metrics, including relative prediction error (PE), mean prediction error (MPE), mean absolute prediction error (MAPE), and the proportion of |PE|% that falls within 20% (F_20_) and 30% (F_30_) (Jaruratanasirikul et al., 2020; Huang et al., 2022). The established evaluation benchmarks were MPE ≤ ±15%, MAPE ≤ 30%, F_20_ > 35%, and F_30_ > 50%.

$$\text{PE }\left(\%\right)=\frac{C_{pred,i}-C_{obs,i}}{C_{obs,i}}$$

$$\text{MPE}=\frac{1}{n}\sum_{i=1}^{n}\left(C_{pred,i}-C_{obs,i}\right)$$

$$\text{MAPE}=\frac{1}{n}\sum_{i=1}^{n}\left|C_{pred,i}\right.\left.{-C}_{obs,i}\right|$$

#### Monte Carlo simulations of dosage regimens

Monte Carlo simulations were conducted utilizing the final population pharmacokinetic model to evaluate the impact of various dosing regimens. The dosing schedules analyzed included: 0.25 g every 6 h, 0.5 g every 6 or 8 h, and 1 g administered every 6, 8, or 12 h. In each scenario, 10,000 virtual patients were simulated to generate profiles of free imipenem concentration over time. The proportion of patients achieving 40% and 100% of the ƒT > MIC against MIC distributions ranging from 0.125 to 16 mg/L for common pathogens treated with imipenem was calculated. A probability of target attainment (PTA) greater than 80% was deemed acceptable, while a PTA exceeding 90% was considered desirable (Jaruratanasirikul et al., 2021).

## Results

### Patient demographics

A total of 142 patients contributing 370 plasma concentration records were included in this study. Data of 120 patients were used to develop the model and 22 patients were used for external validation. Table 1 summarize patient characteristics of modeling group, patients reveal a median age of 72, median weight of 65 kg, and sixty-five percent of patients were males.

**TABLE 1:** Demographics and clinical characteristics.

Characteristics	Modeling group (n = 120)
Gender (male/female), n	78/42
Age, year, (Median [IQR])	72 (68.00, 81.00)
Weight, kg, (Median [IQR])	65 (59.00, 65.33)
DV, μg/mL, (Median [IQR])	1.8 (0.3, 2.775)
INR, (Median [IQR])	1.13 (1.04, 1.27)
PT, s, (Median [IQR])	12.8 (11.80, 14.20)
APTT, s, (Median [IQR])	31.22 (27.60, 36.23)
ALT, IU/L, (Median [IQR])	17.1 (9.60, 32.13)
AST, IU/L, (Median [IQR])	28.7 (18.65, 43.71)
ALP, IU/L, (Median [IQR])	92.3 (65.80, 135.74)
GGT, IU/L, (Median [IQR])	56.3 (29.67, 110.85)
TBIL, μmol/L, (Median [IQR])	15.2 (9.40, 25.70)
DBIL, μmol/L, (Median [IQR])	5.70 (2.70, 12.77)
TP, g/L, (Median [IQR])	56.6 (52.33, 62.53)
ALB, g/L, (Median [IQR])	33 (30.90, 35.64)
GLO, g/L, (Median [IQR])	23.7 (6.00, 15.94)
CREA, μmoI/L, (Median [IQR])	75.00 (52.00, 122.10)
UREA, mmol/L, (Median [IQR])	9.87 (6.00, 15.94)
SCR, μmol/L, (Median [IQR])	232 (150.25, 354.00)
CRP, mg/L, (Median [IQR])	63.8 (26.92, 115.14)
WBC, 10^9^/L, (Median [IQR])	8.6 (5.67, 13.07)
NEUT, 10^9^/L, (Median [IQR])	7.2 (4.30, 11.41)
HGB, g/L, (Median [IQR])	90 (78.00, 104.00)
HCT, %, (Median [IQR])	27.8 (24.13, 31.80)
PLT, 10^9^/L, (Median [IQR])	138 (70.41, 219.00)
PCT, ng/mL, (Median [IQR])	0.593 (0.21, 3.66)
ApoA1, g/L, (Median [IQR])	0.55 (0.40, 0.73)
ApoB, g/L, (Median [IQR])	0.56 (0.43, 0.71)
eGFR, ml·min^-1^/1.73m^2^, (Median [IQR])	86.9 (50.12, 132.20)
CLCR, mL/min, (Median [IQR])	58.9 (35.54, 97.33)
CRRT during imipenem therapy, n (%) of patients	
CRRT	24 (20)

^a^ Values are median (IQR) or n (%). ALB, albumin; ALP, alkaline phosphatase; ALT, alanine aminotransferase; APOA, apolipoprotein A; APOB, apolipoprotein B; APTT, activated partial thromboplastin time; AST, aspartate aminotransferase; CREA, creatinine; CRP, C-reactive protein; CRRT, continuous renal replacement therapy; CLCR, creatinine clearance rate (according to Cockcroft–Gault formulation); DBIL, direct bilirubin; DV, the observed value; GFR, glomerular filtration rate; GGT, gamma-glutamyl transpeptidase; GLO, globulin; HGB, hemoglobin; HCT, hematocrit; INR, international normalized ratio; SCR, serum creatinine; TBIL, total bilirubin; TP, total protein; NEUT, neutrophil count; PCT, procalcitonin; PLT, platelet; PT, prothrombin time; WBC, white blood cell count.

### Population pharmacokinetic modeling

**FIGURE 1:**
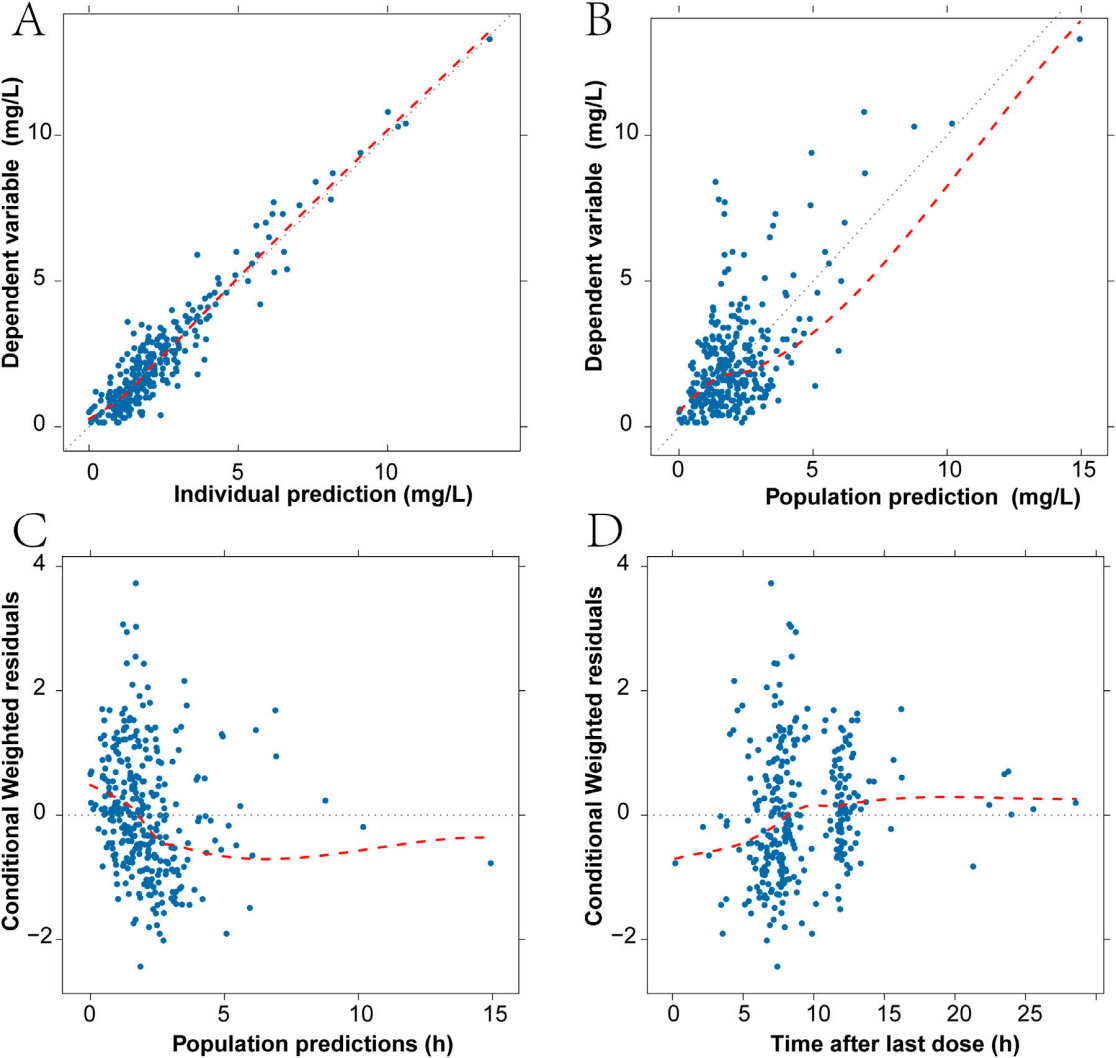
Diagnostic goodness-of-fit plots for the final model. **(A)** Scatter plot of imipenem plasma concentration and individual predicted values. **(B)** Scatter plot of imipenem plasma concentration and population predicted values. **(C)** Scatter plot of conditional weighted residuals and population predicted values. **(D)** Scatter plot of conditional weighted residuals and time from last dose. The black dashed line is the reference line, and the red dashed line is the LOWESS trend line.

The pharmacokinetic profiles of Imipenem were effectively characterized using a two-compartment model with linear elimination. Various residual error models were evaluated, and the additive residual error model assuming a normal distribution was determined to best fit the data. In the final model, the inter-individual variation values for V_c_, Q, and V_p_ were constrained due to inadequate precision in the omega estimates. Through the forward selection process, the covariates CLCR, CRP, WBC, and CRRT were incorporated into the clearance parameter (CL), resulting in reductions in the objective function value (OFV) of 61, 6.02, 4.47, and 6.64, respectively. In the recursive backward elimination approach, WBC, CRRT, and CRP were sequentially removed from CL. After conducting forward inclusion and backward elimination for all covariates, CL was predominantly affected only by CLCR. Supplementary Table S1 provides the summary statistics for the subjects’ covariates selection during the PPK model development. The goodness of fit (GOF) plots for the final model of IMP are illustrated in Figure 1. The observations versus predictions data displayed a consistent distribution along the line of identity. Most conditional weighted residuals (CWRES) fell within the interval of −2 to 2. The final parameter estimates are detailed in Table 2, and both the fixed effects and random variance parameter estimates were determined with high precision. The resulting PPK model for imipenem list as Equations 1-4:

$$CL\;\left(L/h\right)=13.1\times {\left(\frac{CLCR}{71}\right)}^{0.263}\times \mathrm{EXP}\left(\eta CL\right)$$ (1)

$$V_{C}\;\left(L\right)=11.7$$ (2)

$$Q\;\left(L\right)=11.9$$ (3)

$$V_{P}\;\left(L\right)=29.3$$ (4)

**TABLE 2:** Estimated PPK parameters and results of bootstrap evaluation.

Parameters	Final model			Bootstrap	
	Estimate	RSE (%)	Shrinkage (%)	Median	95%CI
CL (L/h)	13.1	4.80	NA	13.3	9.11–14.4
V_c_ (L)	11.7	5.20	NA	11.7	3.41–12.7
Q (L/h)	11.9	24.5	NA	12.1	5.67–18.0
V_p_ (L)	29.3	12.3	NA	30.3	17.5–41.4
θ_5_ (CLCR on CL)	0.263	14.0	NA	0.264	0.196–0.341
Inter-individual variability (IIV)					
ω^2^CL	0.0832	16.7	10.6	0.0802	0.0545–0.111
Residual variability (RSV)					
Additive error	0.575	13.8	8.40	0.572	0.422–0.721

RSE, relative standard error; NA, not applicable; CL, clearance; V_c_, distribution volume of central compartment; Q, inter-compartemental clearance; V_p_, distribution volume of the peripheral compartment; CLCR, creatinine clearance rate.

### Model evaluation

**FIGURE 2:**
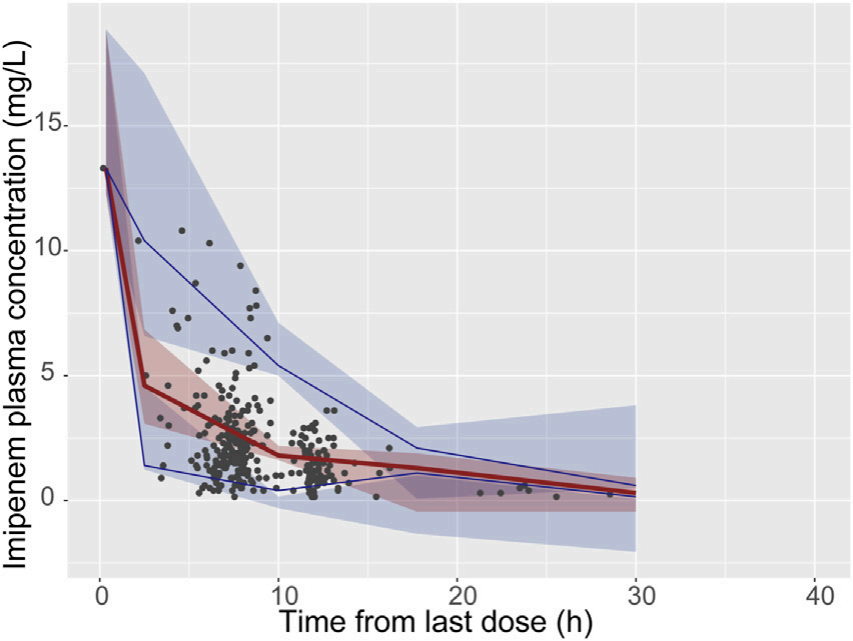
Visual predictive check (VPC) of the final model. The black solid points represent the dependent variable; upper, middle, and lower solid line represent the 95th, 50th and 5th percentiles of the observed concentrations, respectively; The upper, middle, and lower shaded sections represent the 95% confidence interval for the 95th, 50th, and 5th percentiles of the simulated concentrations, respectively.

A total of 967 out of 1,000 (96.7%) successfully converged during the bootstrap evaluation process, indicating a robust stability for the final PPK model. The bootstrap results are presented in Table 2. The medians of the parameter values derived from the bootstrap analysis closely aligned with the final parameters obtained from the original dataset, demonstrating an outstanding prediction capability of the final population model. The pcVPC results illustrated in Figure 2 show that the observed median (denoted by a solid line) and the observed 5th and 95th percentiles (represented by solid lines) are adequately positioned within the simulation-derived prediction intervals (shown as shaded regions). These findings suggest that the final model exhibits a satisfactory level of predictive performance. The external validation produced MPE and MAPE values of 7.6% and 39.4%, respectively. Additionally, the data indicates that F_20_ constitutes 34% of the total, while F_30_ makes up 52.8%, reflecting an acceptable performance level. Overall, the final model demonstrated commendable precision and accuracy, aligning effectively with the previously mentioned evaluation criteria.

### Monte Carlo simulations of dosage regimens

**FIGURE 3:**
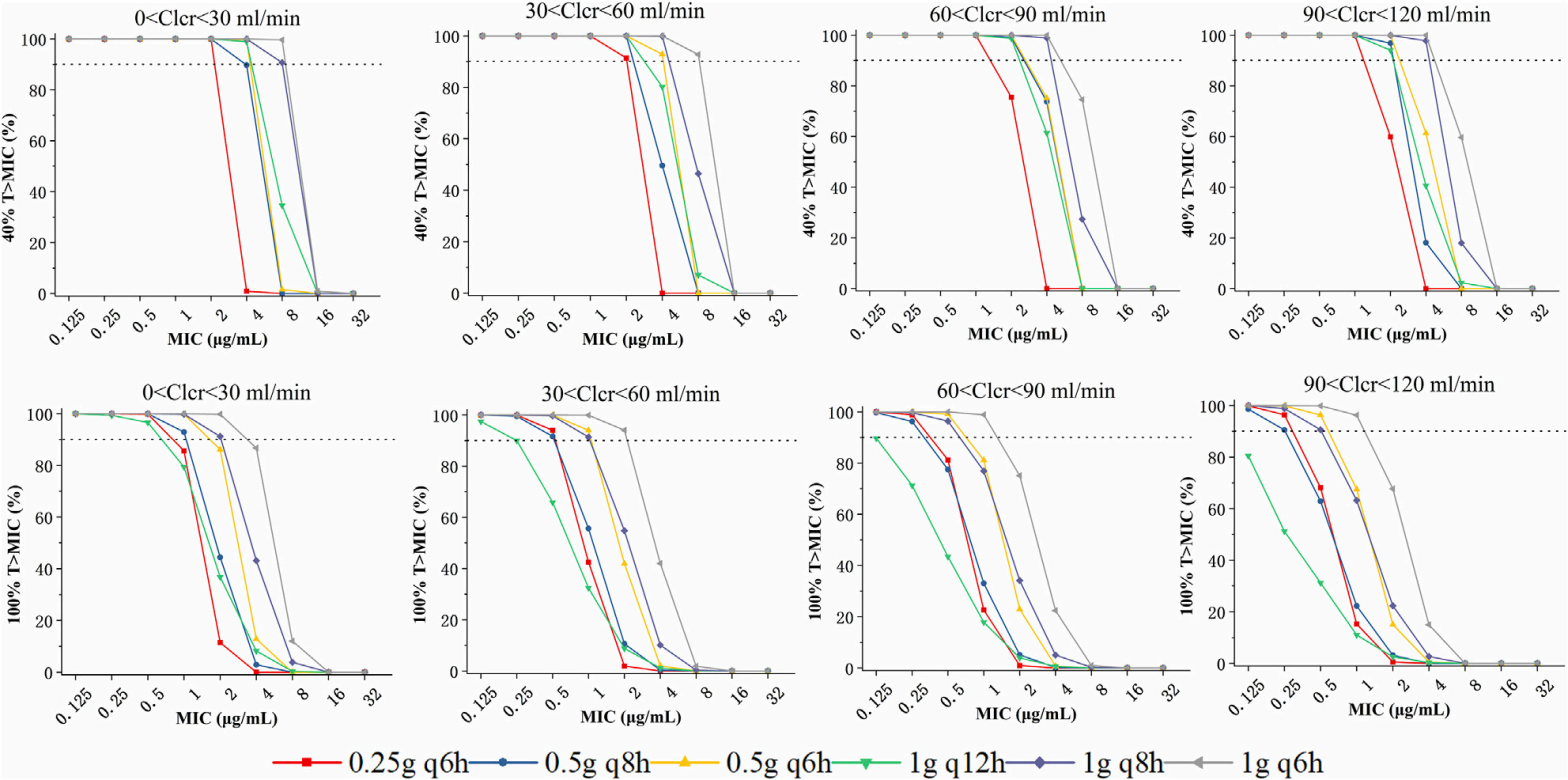
Probability of target attainment (PTA) of various imipenem dose regimens with a target of 40% (upper panels) and 100%T > MIC (lower panels), stratified based on patient renal function:0 <-0, 30–60, 60–90, and 90–120 mL/min.

According to simulated time-dependent concentration data (at steady-state), PTAs (40% or 100% ƒT > MIC) was calculated for the 6 dosage regimens including 250 mg every 6 h, 500 mg every 6 or 8 h, and 1,000 mg administered every 6, 8, or 12 h. The CLCR threshold was utilized to categorize the virtual patients into four groups: 0-30, 30–60, 60–90, and 90–120 mL/min. And the dosing regimens summarized for different CLCR and MIC are listed in Table 3. The PTAs of 40%ƒT > MIC are shown in Figure 3A. At the 40%ƒT > MIC threshold, the pharmacokinetics of all simulated patient treatment adjuncts were sufficient for administration when MIC was ranging from 0.125 to 1 μg/mL for the 6 dosage regimens. For an MIC of 2 μg/mL, PTA was recorded at 75.44% and 59.92% with a dosage of 250 mg administered every 6 h under CLCR of 60–90, 90–120 mL/min, which fell short of the desired 90% PTA, and a higher dose of 500 mg every 8 h is needed. In the case of an MIC of 4 μg/mL, a dosage of 500 mg every 6 h is recommended for patients with a CLCR of 0–60 mL/min, while for those with CLCR greater than 60 mL/min, a dosage of 1,000 mg every 8 h is necessary to achieve over 90% PTA. For an MIC of 8 μg/mL, the only regimen capable of surpassing 90% PTA under a CLCR of 0–60 mL/min is 1,000 mg every 6 h. Conversely, for CLCR greater than 60 mL/min, no regimens meet the threshold for 90% PTA. No feasible imipenem dosing strategy could effectively target highly resistant pathogens with a MIC of 16 mg/L or higher.

**TABLE 3:** Dosage regimens recommended for different pharmacodynamic index based on stratification of CLCR and MIC values.

MIC	40% *ƒ*T > MIC		100% *ƒ*T > MIC	
	CLCR 0–60 mL/min	CLCR 60–120 mL/min	CLCR 0–60 mL/min	CLCR 60–120 mL/min
0.125	0.25 g q6 h		0.25 g q6 h	
0.25				
0.5			0.25 g q6 h	0.5 g q6 h
1			0.5 g q8 h	1 g q6 h
2	0.25 g q6 h	0.5 g q8 h	1 g q6 h	None
4	0.5 g q6 h	1 g q8 h	None	
8	1 g q6 h	None		
16	None			

MIC, minimum inhibitory concentration; CLCR, creatinine clearance rate.

The PTAs for the 100%ƒT > MIC threshold are illustrated in the lower panels of Figure 3. At the 100%ƒT > MIC level, a dosing regimen of 250 mg every 6 h could achieve the 90%PTA under the MIC of 0.125–0.5 μg/mL under a CLCR of 0–60 mL/min. While for CLCR of 60–120 mL/min, it failed to attain the 90% PTA benchmark, and 500 mg every 6 h is required. For an MIC of 1 μg/mL, patients exhibiting a CLCR of 60–120 mL/min require 1,000 mg every 6 h, whereas those with a CLCR of 0–60 mL/min can be managed with a dosage of 500 mg every 8 h. When the MIC reaches 2 μg/mL, it is recommended that patients with a CLCR of 0–60 mL/min receive 1,000 mg every 6 h, while PTAs for other dosing regimens all fell below 86.11%. For MIC levels exceeding 4 μg/mL, all calculated PTAs were less than 86.7%.

To provide a more illustrative depiction of the impact of covariates and dosing regimens on serum imipenem concentrations, the temporal variation of serum imipenem levels across various dosing strategies and differing creatinine clearance rates was simulated, as presented in Supplementary Figures S1, S2. The simulated 97.5th, 50th, and 2.5th percentiles of steady-state concentrations were analyzed alongside 95% confidence intervals to assess variability and ensure robust recommendations for dosing regimens.

## Discussion

This research presents a population pharmacokinetic analysis of imipenem conducted in elderly Chinese patients, including those in critical condition. Significant covariates, such as creatinine clearance, that affect the disposition of imipenem were identified. Understanding their impact on imipenem pharmacokinetics will aid in guiding initial dose adjustments to optimize antibacterial exposure in this demographic.

According to Imani (Imani et al., 2018) and Wu (Wu et al., 2024), younger age has been recognized as a significant risk factor for non-attainment of imipenem concentration targets, which was 1.653-fold higher in patients aged ≤60 years than those aged >60 years. On one hand, this is due to the reduced renal function observed in elderly patients, resulting in a slower clearance rate of imipenem and facilitating the achievement of target levels. However, this situation can also result in elevated imipenem concentrations within the elderly population compared to their younger counterparts, potentially increasing the risk of adverse reactions (Qiao et al., 2022). It's important to note, that not all elderly individuals experience impaired kidney function, and substantial inter-individual variability exists. Consequently, it is essential to investigate the relevant influencing factors associated with imipenem administration in this demographic and to develop tailored dosing regimens rooted in renal function assessments.

Imipenem is a hydrophilic β-lactam antibiotic characterized by a relatively low steady-state volume and minimal protein binding, typically ranging from 10% to 20% (Lafaurie et al., 2023). The two-compartment model emerged as the most effective framework for depicting the concentration-time profile of imipenem, aligning with findings from earlier population pharmacokinetic studies (Dinh et al., 2022; Fratoni et al., 2022). The volume of imipenem in our study was consistent with the values obtained from previous researches (Couffignal et al., 2014). The CL was greater than the values obtained from a previous study (Van Hasselt et al., 2016; Bai et al., 2024), which may be due to the higher GFR of our patients. The renal function calculated by CLCR was identified as the most significant covariate during the covariate model development process. This finding aligns with expectations, considering that a substantial proportion of imipenem is eliminated through renal excretion. It's worth noting that CRRT was incorporated in full regression model. During the treatment and sampling period, CRRT was administered to 39 patients, impacting the pharmacokinetic population model. Nevertheless, the incorporation of this variable only decreased the OFV to 5.81, leading to its exclusion from the final model based on the recursive backward elimination criterion. CLCR value was derived from serum creatinine, which can indirectly reflect the clearing effect of CRRT. Therefore, the influence of CRRT factors was indirectly reflected in the model during the optimization of CLCR (Chen et al., 2020).

For β-lactam antibiotics, the pharmacodynamic indicator of clinical effectiveness and the potential for microbial resistance development to imipenem is typically represented by the percentage of unbound drug concentrations sustained above the minimum inhibitory concentration (MIC) of the pathogen (ƒT > MIC). A threshold of at least 40% is generally advised (Abdul-Aziz et al., 2022). For patients in critical condition, the pharmacodynamic target for β-lactams is regarded as 100% ƒT > MIC (Li and Xie, 2019). Consequently, in this study, we conducted a dosing simulation to assess the likelihood of achieving target levels of 40% and 100% T > MIC by employing different dosing regimens and considering the renal functions of elderly patients during the administration of imipenem. Besides, the temporal variation of serum imipenem levels across differing CLCR distribution was simulated, as presented in Supplementary Figure S1 alongside 95% confidence intervals to assess variability and ensure robust recommendations for dosing regimens. According to the Monte Carlo simulation results, the recommended drug administration regimen under different conditions was further summarized and listed in Table 3, which can provide reference for rational use of imipenem in clinical practice.

This research presents several limitations that warrant consideration. Firstly, due to the clinical practice surrounding therapeutic drug monitoring focusing primarily on trough concentration points, the samples we gathered were predominantly comprised of sparse sampling intervals, which could potentially impact the accuracy of the derived population pharmacokinetic models. Nevertheless, our pharmacokinetic models showed a reasonable correlation with existing literature, indicating their suitability for dose simulation purposes. Secondly, the study was conducted on a limited cohort of participants, whose individual characteristics, such as body weight (ranging from 35 to 93.5 kg), may vary significantly. Lastly, the Cockcroft-Gault equation employed for estimating creatinine clearance may inadvertently overstate the influence of age on creatinine excretion among elderly patients.

## Conclusion

Our research established a population pharmacokinetic model based on the plasma concentration of imipenem in elderly Chinese patients. Simulations derived from this validated model endorse recommendations for adjusting imipenem dosages in renal-impaired subpopulations, tailored to renal function, in order to achieve adequate probability of target attainment while keeping drug exposure within safe limits. Additionally, clinicians can leverage these simulation outcomes to determine optimal imipenem administration strategies for diverse patients, ensuring maximum clinical efficacy, particularly for those with elevated CLCR rates and MIC, who may necessitate higher doses or more frequent dosing intervals.

## Data Availability

The original contributions presented in the study are included in the article/Supplementary Material, further inquiries can be directed to the corresponding author.

## References

[B1] Abdul-Aziz M. H. Brady K. Cotta M. O. Roberts J. A. (2022). Therapeutic drug monitoring of antibiotics: defining the therapeutic range. Ther. Drug Monit. 44, 19–31. 10.1097/FTD.0000000000000940 34750338

[B2] Abdulla A. Ewoldt T. M. J. Purmer I. M. Muller A. E. Gommers D. Endeman H. (2021). A narrative review of predictors for β-lactam antibiotic exposure during empirical treatment in critically ill patients. Expert Opin. Drug Metab. Toxicol. 17, 359–368. 10.1080/17425255.2021.1879049 33463382

[B3] Ahamadi M. Largajolli A. Diderichsen P. M. De Greef R. Kerbusch T. Witjes H. (2019). Operating characteristics of stepwise covariate selection in pharmacometric modeling. J. Pharmacokinet. Pharmacodyn. 46, 273–285. 10.1007/s10928-019-09635-6 31020450

[B4] Bai J. Wen A. Li Z. Li X. Duan M. (2024). Population pharmacokinetics and dosing optimisation of imipenem in critically ill patients. Eur. J. Hosp. Pharm. 31, 434–439. 10.1136/ejhpharm-2022-003403 36948580 PMC11347199

[B5] Benson J. M. (2017). Antimicrobial pharmacokinetics and pharmacodynamics in older adults. Infect. Dis. Clin. North Am. 31, 609–617. 10.1016/j.idc.2017.07.011 29079151

[B6] Bergstrand M. Hooker A. C. Wallin J. E. Karlsson M. O. (2011). Prediction-corrected visual predictive checks for diagnosing nonlinear mixed-effects models. AAPS J. 13, 143–151. 10.1208/s12248-011-9255-z 21302010 PMC3085712

[B7] Byon W. Smith M. K. Chan P. Tortorici M. A. Riley S. Dai H. (2013). Establishing best practices and guidance in population modeling: an experience with an internal population pharmacokinetic analysis guidance. CPT Pharmacomet. Syst. Pharmacol. 2, e51. 10.1038/psp.2013.26 PMC6483270 23836283

[B8] Chen W. Zhang D. Lian W. Wang X. Du W. Zhang Z. (2020). Imipenem population pharmacokinetics: therapeutic drug monitoring data collected in critically ill patients with or without extracorporeal membrane oxygenation. Antimicrob. Agents Chemother. 64, e00385. 10.1128/AAC.00385-20 32253220 PMC7269497

[B9] Couffignal C. Pajot O. Laouénan C. Burdet C. Foucrier A. Wolff M. (2014). Population pharmacokinetics of imipenem in critically ill patients with suspected ventilator‐associated pneumonia and evaluation of dosage regimens. Br. J. Clin. Pharmacol. 78, 1022–1034. 10.1111/bcp.12435 24903189 PMC4243876

[B10] Dinh T. D. Nguyen H. N. Le B. H. Nguyen T. T. T. Nguyen H. T. L. (2022). Population-based pharmacokinetics and dose optimization of imipenem in Vietnamese critically-ill patients. Infect. Drug Resist. 15, 4575–4583. 10.2147/IDR.S373348 36003989 PMC9393097

[B11] Fratoni A. J. Mah J. W. Nicolau D. P. Kuti J. L. (2022). Imipenem/cilastatin/relebactam pharmacokinetics in critically ill patients with augmented renal clearance. J. Antimicrob. Chemother. 77, 2992–2999. 10.1093/jac/dkac261 35906810

[B12] Gomez D. S. Sanches-Giraud C. Silva C. V. Oliveira A. M. R. R. Da Silva J. M. Gemperli R. (2015). Imipenem in burn patients: pharmacokinetic profile and PK/PD target attainment. J. Antibiot. (Tokyo) 68, 143–147. 10.1038/ja.2014.121 25227503

[B13] Huang W. Zheng Y. Huang H. Cheng Y. Liu M. Chaphekar N. (2022). External evaluation of population pharmacokinetic models for voriconazole in Chinese adult patients with hematological malignancy. Eur. J. Clin. Pharmacol. 78, 1447–1457. 10.1007/s00228-022-03359-2 35764817

[B14] Imani S. Buscher H. Day R. Gentili S. Jones G. R. D. Marriott D. (2018). An evaluation of risk factors to predict target concentration non-attainment in critically ill patients prior to empiric β-lactam therapy. Eur. J. Clin. Microbiol. Infect. Dis. Off. Publ. Eur. Soc. Clin. Microbiol. 37, 2171–2175. 10.1007/s10096-018-3357-9 30120647

[B15] Jaruratanasirikul S. Boonpeng A. Nawakitrangsan M. Samaeng M. (2021). NONMEM population pharmacokinetics and Monte Carlo dosing simulations of imipenem in critically ill patients with life-threatening severe infections during support with or without extracorporeal membrane oxygenation in an intensive care unit. Pharmacother. J. Hum. Pharmacol. Drug Ther. 41, 572–597. 10.1002/phar.2597 34080708

[B16] Jaruratanasirikul S. Vattanavanit V. Wongpoowarak W. Nawakitrangsan M. Samaeng M. (2020). Pharmacokinetics and Monte Carlo dosing simulations of imipenem in critically ill patients with life-threatening severe infections during support with extracorporeal membrane oxygenation. Eur. J. Drug Metab. Pharmacokinet. 45, 735–747. 10.1007/s13318-020-00643-3 32886347 PMC7471576

[B17] Klotz U. (2009). Pharmacokinetics and drug metabolism in the elderly. Drug Metab. Rev. 41, 67–76. 10.1080/03602530902722679 19514965

[B18] Koucheki R. Dowling K. I. Patel N. R. Matsuura N. Mafeld S. (2021). Characteristics of imipenem/cilastatin: considerations for musculoskeletal embolotherapy. J. Vasc. Interv. Radiol. JVIR 32, 1040–1043.e1. 10.1016/j.jvir.2021.04.006 34210475

[B19] Lafaurie M. Burdet C. Hammas K. Goldwirt L. Berçot B. Sauvageon H. (2023). Population pharmacokinetics and pharmacodynamics of imipenem in neutropenic adult patients. Infect. Dis. Now. 53, 104625. 10.1016/j.idnow.2022.09.020 36174960

[B20] Li S. Xie F. (2019). Population pharmacokinetics and simulations of imipenem in critically ill patients undergoing continuous renal replacement therapy. Int. J. Antimicrob. Agents 53, 98–105. 10.1016/j.ijantimicag.2018.10.006 30626495

[B21] Mangoni A. A. Jackson S. H. D. (2004). Age‐related changes in pharmacokinetics and pharmacodynamics: basic principles and practical applications. Br. J. Clin. Pharmacol. 57, 6–14. 10.1046/j.1365-2125.2003.02007.x 14678335 PMC1884408

[B22] Medellín-Garibay S. E. Romano-Aguilar M. Parada A. Suárez D. Romano-Moreno S. Barcia E. (2022). Amikacin pharmacokinetics in elderly patients with severe infections. Eur. J. Pharm. Sci. Off. J. Eur. Fed. Pharm. Sci. 175, 106219. 10.1016/j.ejps.2022.106219 35618200

[B23] Papp-Wallace K. M. Endimiani A. Taracila M. A. Bonomo R. A. (2011). Carbapenems: past, present, and future. Antimicrob. Agents Chemother. 55, 4943–4960. 10.1128/AAC.00296-11 21859938 PMC3195018

[B24] Pietroski N. A. Graziani A. L. Lawson L. A. Bland J. A. Rogers J. D. MacGregor R. R. (1991). Steady-state pharmacokinetics of intramuscular imipenem-cilastatin in elderly patients with various degrees of renal function. Antimicrob. Agents Chemother. 35, 972–975. 10.1128/AAC.35.5.972 1854179 PMC245138

[B25] Qiao W. Chang C. Wang Q. Cao X. Zhang X. (2022). Imipenem cilastatin sodium-associated thrombocytopenia in an older patient: a case report and literature review. Int. J. Clin. Pharmacol. Ther. 60, 358–363. 10.5414/CP204215 35652550

[B26] Shen M. Machado S. G. (2017). Bioequivalence evaluation of sparse sampling pharmacokinetics data using bootstrap resampling method. J. Biopharm. Stat. 27, 257–264. 10.1080/10543406.2016.1265543 27906608

[B27] Van Hasselt J. G. C. Rizk M. L. Lala M. Chavez‐Eng C. Visser S. A. G. Kerbusch T. (2016). Pooled population pharmacokinetic model of imipenem in plasma and the lung epithelial lining fluid. Br. J. Clin. Pharmacol. 81, 1113–1123. 10.1111/bcp.12901 26852277 PMC4876184

[B28] Wu Y. Lu Z. Liang P. Zhu H. Qi H. Zhang H. (2024). Relationship of imipenem therapeutic drug monitoring to clinical outcomes in critically ill patients: a retrospective cohort study. Naunyn. Schmiedeb. Arch. Pharmacol. 397, 4791–4798. 10.1007/s00210-023-02909-4 38153513

[B29] Zhanel G. G. Wiebe R. Dilay L. Thomson K. Rubinstein E. Hoban D. J. (2007). Comparative review of the carbapenems. Comp. Rev. Carbapenems Drugs 67, 1027–1052. 10.2165/00003495-200767070-00006 17488146

