# Corrigendum: Population pharmacokinetics and dosing optimization of imipenem in Chinese elderly patients

**DOI:** 10.3389/fphar.2025.1565995

**Published:** 2025-02-14

**Authors:** Jing Wang, Qiu Fang, Xuemei Luo, Lu Jin, Huaijun Zhu

**Affiliations:** ^1^ Department of Pharmacy, Nanjing Drum Tower Hospital, Nanjing, Jiangsu, China; ^2^ Department of Pharmacy, Nanjing Drum Tower Hospital Clinical College of Nanjing University of Chinese Medicine, Nanjing, Jiangsu, China

**Keywords:** imipenem, population pharmacokinetics, dosing optimization, elderly patients, Monte Carlo

In the published article, there was an error in **Affiliation 1**. Instead of “Department of Pharmacy, The Affiliated Nanjing Drum Tower Hospital of Nanjing University Medical School, Nanjing, Jiangsu, China,” it should be “Department of Pharmacy, Nanjing Drum Tower Hospital, Nanjing, Jiangsu, China.”

In the published article, there was an error regarding the **Affiliation** of “Huaijun Zhu”. As well as having affiliation 1, they should also have “Department of Pharmacy, Nanjing Drum Tower Hospital Clinical College of Nanjing University of Chinese Medicine, Nanjing, Jiangsu, China.”

The authors apologize for these errors and state that these do not change the scientific conclusions of the article in any way. The original article has been updated.

