# Supplementary material for: Population pharmacokinetics and dosing optimization of imipenem in Chinese elderly patients
Source: Front Pharmacol. 2025 Jan 9;15:1524272. doi: 10.3389/fphar.2024.1524272 (PMC11754279; doi:10.3389/fphar.2024.1524272)
Supplement: Supplementary file 1 [file DataSheet1.docx]

**Table S1.** Summary of forward inclusion and backward elimination during model building

| Model No. | | Model description | OFV | △OFV | | *P* Value | | Decision | |
| --- | --- | --- | --- | --- | --- | --- | --- | --- | --- |
| Forward inclusion | | |  | |  | |  | |  |
| 1 | Base model | | 434.6 | | / | | / | | / |
| 2 | Add CLCR on CL in model 1 | | 373.6 | | 61 | | <0.001 | | Accept |
| 3 | Add CRP on CL in model 2 | | 367.58 | | 6.02 | | <0.05 | | Accept |
| 4 | Add WBC on CL in model 3 | | 363.11 | | 4.47 | | <0.05 | | Accept |
| 5 | Add CRRT on CL in model 4 | | 356.47 | | 6.64 | | <0.01 | | Accept |
| Backward elimination | | |  | |  |  | | |  |
| 6 | Remove WBC on CL from model 5 | | 362.89 | | 6.42 | | >0.01 | | Accept |
| 7 | Remove CRRT on CL from model 6 | | 367.58 | | 4.69 | | >0.01 | | Accept |
| 8 | Remove CRP on CL from model 7 | | 373.6 | | 6.02 | | >0.01 | | Accept |
| 9 | Remove CLCR on CL from model 8 | | 434.6 | | 61 | | <0.001 | | Reject |


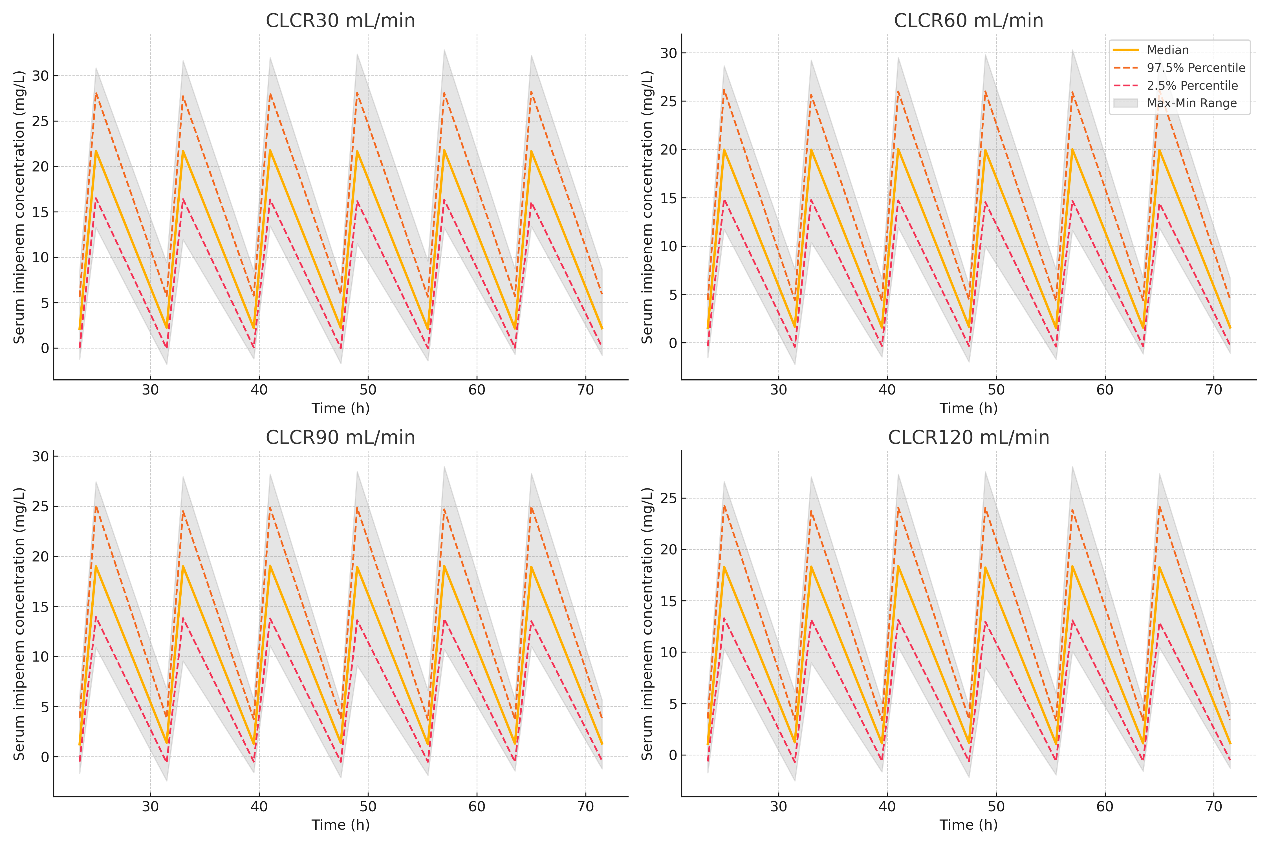
**Figure S1** The temporal variation of serum imipenem concentrations at different levels of creatinine clearance under a fixed dosing regimen of 500 mg every 8 hours.
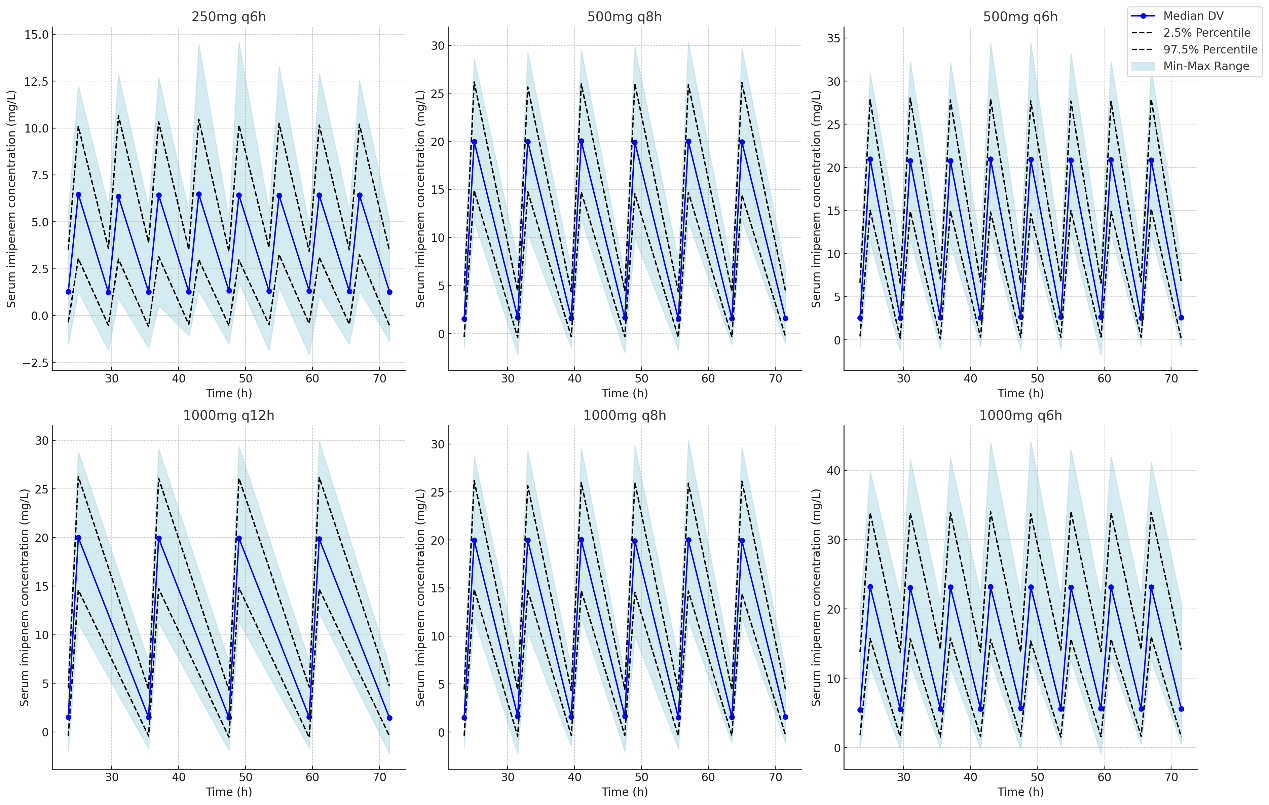


**Figure S2** The temporal variation of serum imipenem concentrations at different levels of dosing regimen under a fixed creatinine clearance of 60 mL/min.
